# Structural analysis of Si-doped amorphous In_2_O_3_ based on quantum beam measurements and computer simulations

**DOI:** 10.1038/s41598-025-20384-0

**Published:** 2025-10-21

**Authors:** Yuta Shuseki, Akihiko Fujiwara, Nobuhiko Mitoma, Takio Kizu, Toshihide Nabatame, Kazuhito Tsukagoshi, Yohei Onodera, Atsunobu Masuno, Koji Ohara, Shinji Kohara

**Affiliations:** 1 Graduate School of Engineering, Kyoto University, Kyoto, 615-8520 Japan; 2 Center for Basic Research on Materials, National Institute for Materials Science (NIMS), Tsukuba, Ibaraki 305-0047 Japan; 3 School of Engineering, Kwansei Gakuin University, Sanda, Hyogo 669-1330 Japan; 4 Research Center for Materials Nanoarchitectonics (MANA), National Institute for Materials Science (NIMS), Tsukuba, Ibaraki 305-0044 Japan; 5 Faculty of Materials for Energy, Shimane University, Matsue, 690-8504 Shimane Japan; 6 Diffraction and Scattering Division, Synchrotron Radiation Research Institute (JASRI), Sayo-gun, Hyogo, 679–5198 Japan

**Keywords:** Amorphous indium oxide, X-ray diffraction, Molecular dynamics simulation, Reverse Monte Carlo, Topological analysis, Thermal stability, Mechanical properties, Electronic devices

## Abstract

The structural properties and thermal stability of Si-doped amorphous indium oxide (ISO) were investigated via experimental characterization and computational modeling techniques. The total structure factors, *S*(*Q*), and reduced pair distribution functions, *G*(*r*), were calculated for both annealed and pristine ISO samples, revealing the distinct structural features induced by Si doping and thermal treatment. Although the pristine ISO samples exhibited halo patterns indicative of an amorphous structure, annealing at 600 °C led to pronounced Bragg peaks, suggesting that the sample was crystallized. However, an ISO with a higher Si content (20 at%) retained its amorphous structure even after annealing, highlighting the role of Si-doping in enhancing the thermal stability. Classical molecular dynamics–reverse Monte Carlo simulations were employed to elucidate the structure of pristine ISO samples, revealing good agreement with the experimental data. Furthermore, the partial structure factors, *S*_*ij*_(*Q*), and partial pair distribution functions, *g*_*ij*_(*r*) demonstrate the influence of Si doping on atomic correlations and density changes in the ISO. Polyhedral connectivity analysis suggests that the fraction changes of edge sharing due to Si doping affect the thermal stability of ISO and that SiO_4_ tetrahedra play a crucial role in inhibiting crystallization.

**Supplementary Information:**

The online version contains supplementary material available at 10.1038/s41598-025-20384-0.

## Introduction

Energy consumption has reached unprecedented levels owing to the proliferation of devices with semiconductors connected to daily applications (Internet of Things), and computers are being used in various situations. To address these energy problems, thin films of metal oxide semiconductors and conductors are garnering attention^1,2^. In particular, thin films of indium oxide (In_2_O_3_) are widely recognized for their low electrical resistance (*ρ *= ~10^–4^ Ωcm) as a conductor and large band gap (*E*_g_ = 3.36) as a semiconductor^3–5^. In addition, these thin-film semiconductors exhibit high carrier mobility and high transparency within the visible light range, making them suitable as both conducting electrodes and semiconductor active layers^6,7^. Historically, expensive single-crystal Si has been used as a channel material for thin-film transistors (TFTs). In contrast, employing cheaper polycrystalline or amorphous Si (*a*-Si) results in a significant decrease in carrier mobility, leading to poor performance^8^. Many studies have focused on amorphous oxide semiconductors, highlighting the high mobility and transparency of materials such as amorphous In–Zn–O (*a*-IZO)^9,10^ and amorphous In–Ga–Zn–O (*a*-IGZO)^11–13^. Although *a*-IGZO exhibits higher electron mobility than *a*-Si TFTs, the structural stability of *a*-IGZO is lacking. Conversely, amorphous Si-doped In_2_O_3_, amorphous In–Si–O (*a*-ISO), has been reported to exhibit higher structural stability than *a*-IGZO^14–17^.

High-energy X-ray diffraction (HEXRD)^18^ is considered to be a suitable method for understanding the atomic structures of various materials, including crystals^19,20^ liquids^21,22^ and amorphous materials^23,24^. Particularly for liquids and amorphous materials lacking long-range structural information, HEXRD over a wide *Q* range is essential for obtaining accurate pair distribution functions, *g*(*r*)^25^. In addition, the combination of quantum beam measurements and computer simulations is a well-known approach for elucidating atomic structures in greater detail. Classical molecular dynamics (CMD) and density functional theory (DFT) are commonly used for modeling inorganic materials^26,27^ offering methods for generating three-dimensional structures and understanding various features such as atomic distances, bond angles, and electronic states. Previous studies have employed simulation techniques to investigate *a*-In_2_O_3_^28,29^. Alternatively, the reverse Monte Carlo (RMC) method can be used to generate structures based on experimental data obtained from quantum beam measurements. Numerous studies have utilized RMC modeling for materials such as SiO_2_ glass^30^ Ge_2_Se_2_ glass^31^ and amorphous alloys^32^.

It is suggested from previous reports that Si-doped enhances the structural stability of amorphous In_2_O_3_, but the reason for this phenomenon is not well understood. The structural change of InO_*x*_–InO_*x*_ induced by Si-doping likely contributes to the structural stability. In particular, the structural comparison between Si-doped In_2_O_3_ and crystalline In_2_O_3_ is a key point to elucidate the structural stabilization. In this study, we aimed to clarify the thermal stability of Si-doped ISO by utilizing structural models derived from CMD–RMC modeling based on data from quantum beam measurements. Specifically, we attempted to understand how the structural changes induced by Si doping affected the thermal stability.

## Experimental and simulation procedures

### Sample preparation

ISO thin films were formed on a quartz substrate (100 mm×100 mm) covered by a poly (methyl methacrylate) resist using a DC magnetron sputtering system (Shibaura Mechatronics, CFS-4EP-LL i-Miller) at room temperature. The three-inch sputtering targets composed of In_2_O_3_ and SiO_2_ with Si concentrations, Si/(In + Si), of 0, 2, 7, 11, and 20 at% were used. The sputtering targets and substrates in the sputtering system were 160 mm apart. DC sputtering was performed at a power of 200 W under an Ar and O_2_ mixed atmosphere with an Ar: O_2_ gas flow ratio of 1:1 at a total pressure of 0.25 Pa^17^. After the thin films were formed, they were removed from the substrate by soaking in acetone. The removed thin films were encapsulated in glass capillaries. The as-grown samples and those annealed at 600 °C in a muffle furnace were used for the HEXRD measurements.

### HEXRD

The HEXRD measurements were conducted on the BL04B2 beamline^33^ of SPring-8 in Japan, utilizing an energy of 61.4 keV. The obtained data were normalized, including adjustments for polarization, absorption, background, and Compton scattering, using the original software^33^ of the BL04B2 beamline. Subsequently, all data were normalized to Faber–Ziman total structure factors, *S*(*Q*)^34^.

1 $$\:S\left(Q\right)=1+\frac{1}{|<w\left(Q\right)>{|}^{2}}\sum\:_{\alpha\:}\sum\:_{\beta\:}{c}_{\alpha\:}{c}_{\beta\:}{w}_{\alpha\:}^{*}\left(Q\right){w}_{\beta\:}\left(Q\right)[{S}_{\alpha\:\beta\:}\left(Q\right)-1],$$

where *Q* is the magnitude of the scattering vector, *c*_*α*_ is the atomic fraction of chemical species *α*, *w*_*α*_ is the atomic form factor with dispersion terms for chemical species *α* and is, in general, a complex number, *S*_*αβ*_(*Q*) is the partial structure factor, and |<*w*(*Q*)>| is calculated according to the method described in Eq. (2).

2 $$\:|<w\left(Q\right){>|}^{2}=\sum\:_{\alpha\:}\sum\:_{\beta\:}{c}_{\alpha\:}{c}_{\beta\:}{w}_{\alpha\:}^{*}\left(Q\right){w}_{\beta\:}\left(Q\right).$$

The reduced pair distribution function, *G*(*r*), was obtained using the Fourier transform of *S*(*Q*).

3 $$\:G\left(r\right)=\frac{2}{\pi\:}{\int\:}_{{Q}_{\text{m}\text{i}\text{n}}}^{{Q}_{\text{m}\text{a}\text{x}}}Q\left[S\left(Q\right)-1\right]\text{sin}\left(Qr\right)dQ.\:$$

Hence, Eq. (3) can be converted as follows.

4 $$\:g\left(r\right)=\frac{G\left(r\right)}{4\pi\:r{\rho\:}_{0}}+1,$$

5 $$\:g\left(r\right)=\frac{\rho\:\left(r\right)}{{\rho\:}_{0}},$$

where *ρ*_0_ = N/V, N/V is the number of atoms in the volume V and *ρ*(*r*) is a density at distance *r*. The number density can be calculated from the value of the minimum of *G*(*r*)/4π*r* in Eq. (6)^35^.

6 $$\:\left|{\left\{\frac{G\left(r\right)}{4\pi\:r}\right\}}_{min}^{r\ge\:{r}_{min}}\right|=|\rho\:\left(r\right)-{\rho\:}_{0}|\approx\:{\rho\:}_{0}.$$

The accuracy of this method using SiO_2_ glass is summarized in Fig. S1. The density of SiO_2_ glass is 2.20 g/cm^3^, while our estimated value is 2.27 g/cm^3^. The error of the density was estimated to be 3.1%.

Table 1 summarizes the target concentration and the estimated density data from *G*(*r*) for ISO.

**Table 1:** Composition and estimated density of Si-doped amorphous In_2_O_3_.

	Targeted concentrations of ISO			Estimated density from G(*r*)
	In	Si	O	
ISO0 (In_2_O_3_)	2.00	0	3.00	6.28
ISO2	39.00	1.00	60.00	6.18
ISO7	36.84	2.63	60.53	6.09
ISO11	34.91	4.24	60.85	5.92
ISO20	30.58	7.85	61.57	5.84

### Structural modeling and analysis

Computational structural modeling was performed using CMD and RMC modeling. The generation of the initial configurations, CMD, was employed with the Born–Mayer–Huggins (BMH)^36^ force field utilizing the potential parameters reported by Utsuno et al.^37^. In addition, we used the SiO_2_ potential parameter reported by Zhen et al.^38^. The equations and parameters are detailed in Eq. (7) and are listed in Table 2. Although we evaluated various parameters^37,39^ in Fig. S2, none showed significant differences. We ultimately chose the potential parameters of Utsuno et al. because their data were based on amorphous thin In_2_O_3_ films.

7 $$\:{\varPhi\:}_{ij}=\frac{{e}^{2}}{4\pi\:{\epsilon\:}_{0}}\frac{{Z}_{i}{Z}_{j}}{{r}_{ij}}+{A}_{ij}\text{exp}\left(-{B}_{ij}{r}_{ij}\right),$$

**Table 2:** Potential parameter for classical molecular dynamics simulation of BMH.

	*A*_*ij*_ / J	*B*_*ij*_ / Å^–1^
In–O	2.47 × 10^–14^	5.50
In–In	5.30 × 10^–16^	3.00
O–O	2.71 × 10^–16^	3.00
Si–O	1.01 × 10^–14^	6.06

where *e* is the elementary charge, *r*_*ij*_ is the distance between *i* and *j* atoms, *Z*_*i*_ is the electronic charge of the *i* atom (*Z*_In_ = + 3.0, *Z*_O_ = –2.0, *Z*_Si_ = + 4.0), and *A*_*ij*_ and *B*_*ij*_ are parameters for the *i*–*j* pair. Periodic boundary conditions were employed in the simulation and long-range Coulombic interactions were calculated using the Ewald method. When combining the potentials derived from different sources, variations in the parameters can affect the results, and thus, we compared the *S*(*Q*) and *G*(*r*) curve obtained using CMD and CMD–RMC to evaluate the effect of mixing the potentials (Fig. S3). No unnatural changes or unrealistic structures are observed with increasing amounts of Si doping. Furthermore, upon comparing the coordination numbers of the structures obtained using the CMD and CMD–RMC models (Table S1), the difference in the most important In–O coordination number is extremely small (approximately 1%). Additionally, the differences in the coordination numbers of In–In and O–O are limited to approximately 3% and 1%, respectively. Even the differences in the coordination numbers of ISO0, which does not contain Si, and ISO2–20, which contain Si, are minimal, confirming that the influences of the differences in the potentials on the structure are limited. These results suggest that the framework structure is determined via CMD in this study, and modifications via RMC are mainly limited to the fine tuning of the long-range structure. Therefore, the topological characteristics of the amorphous structure remain essentially unchanged. A cubic cell contains approximately 10,000 atoms arranged randomly (In: 4000, O: 6000 for ISO0, In: 3900, Si: 100, O: 6050 for ISO2, In:3684, Si:263, O: 6052 for ISO7, In:3492, Si:424, O:6086 for ISO11, and In:3058, Si:785, O:6157 for ISO20) in the initial configuration. CMD simulations were performed in the NVT ensemble after annealing for 100 ps at 1000 K, cooling at a rate of 97 K/ps from 1000 to 300 K, and equilibration at 300 K for 10 ps. The CMD simulations were conducted using the LAMMPS program^40^. Following the CMD simulation, RMC modeling utilizing the RMC + + code^41^ was used to refine the structure based on the HEXRD experimental data.

The coordination numbers and polyhedral connectivity were computed using the original program with the first coordination distances of In–In: 4.50 Å, In–O: 2.8 Å, Si–O: 1.9 Å and O–O: 4.00 Å, respectively. In addition, the king ring size distribution was calculated using the R.I.N.G.S. code^42^. The reduced pair distribution functions, *G*(*r*), of the crystalline In_2_O_3_ was calculated using PDFgui^43^.

## Results and discussion

### High energy X-ray diffraction data of ISO

**Fig. 1:**
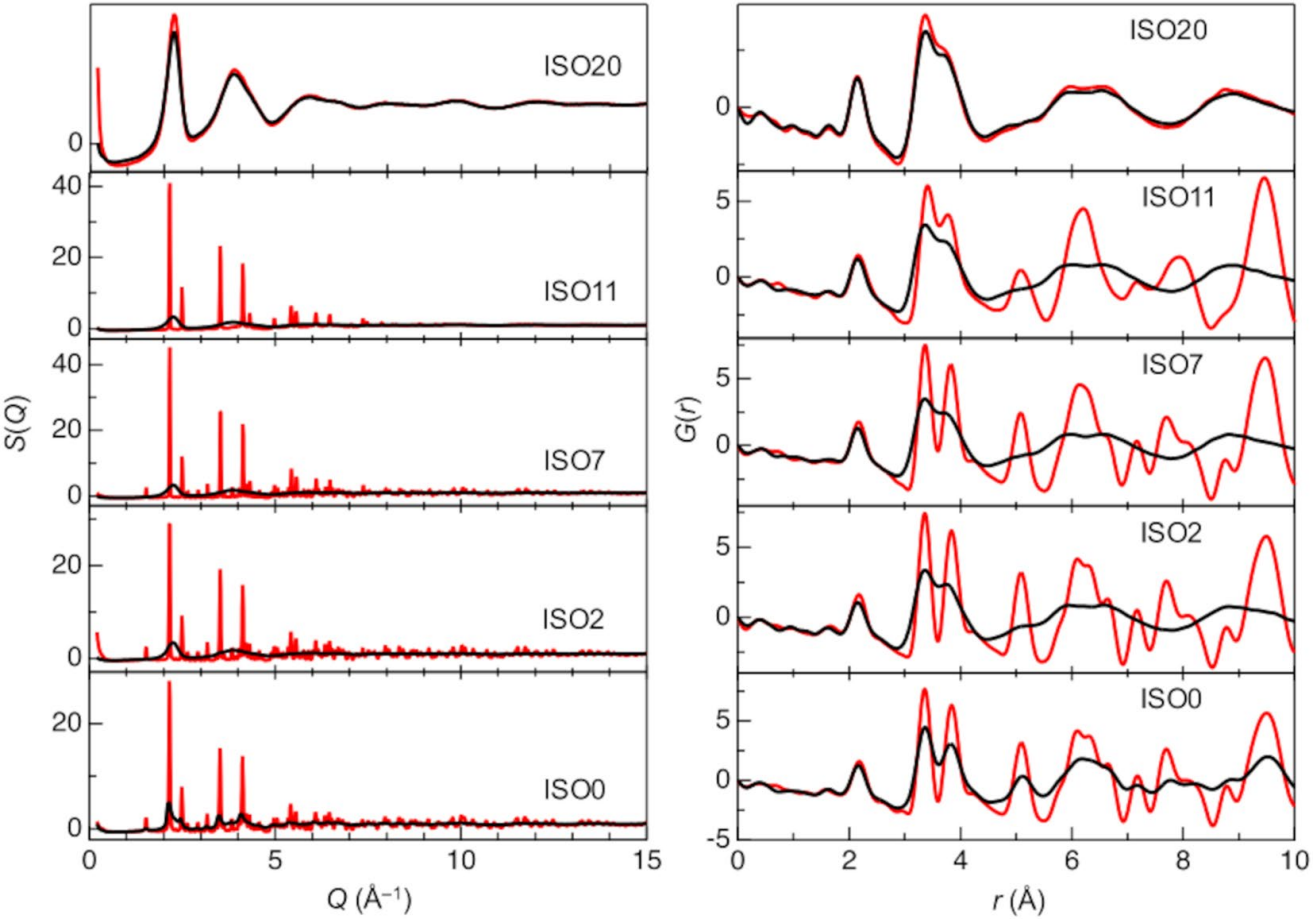
X-ray total structure factors, *S*(*Q*), and reduced pair distribution functions, *G*(*r*), of pristine and annealed ISO0, 2, 7, 11, and 20. Black line, pristine samples; red line, annealed samples.

Figure 1 (left) shows the total structure factors, *S*(*Q*), of both annealed (red line) and pristine (black line) ISO0, 2, 7, 11, and 20. The pristine ISO samples, except ISO0, exhibited a halo pattern, which is characteristic of an amorphous structure. This behavior suggests that Si doping more readily induces the formation of an amorphous structure in the ISO. In contrast, ISO0, 2, 7, and 11 annealed at 600 °C exhibited pronounced Bragg peaks, indicating crystallization of the samples due to thermal treatment. However, only ISO20 retained its amorphous structure at temperatures above 600 °C, suggesting that over 20 at% Si doping is necessary to maintain an amorphous structure during the thermal treatment of ISO. The reduced pair distribution functions, *G*(*r*), of both annealed and pristine ISO0, 2, 7, 11, and 20 are shown in Fig. 1 (right). While all the peaks are located at similar *r* positions, the annealed samples exhibit peaks attributed to the improved periodicity. Consequently, the *G*(*r*) around long ranges (over 5.00 Å) of annealed ISO0, 2, 7, and 11 (crystalline) shows more obvious peaks compared to the amorphous ones. These peaks reveal atomic correlations, such as In–O (2.10 Å) and In–In/O–O (ranging from 3.20 to 3.90 Å). Figure S4, in which *G*(*r*) was calculated using crystal structures from previous research^44,45^ shows that all samples transitioned to cubic In_2_O_3_ after annealing, in contrast to the cubic, trigonal, and orthorhombic *G*(*r*) structures of crystalline In_2_O_3_. Typically, Si atoms form SiO_4_ tetrahedra in amorphous samples, with an Si–O distance of approximately 1.60 Å. However, the *G*(*r*) data for all samples did not clearly exhibit Si–O peaks because X-rays are scattered by electron clouds, so the scattering ability varies depending on the atomic species (number of electrons). We calculated the approximate X-ray weighting factors, *W*_*ij*_, using Eq. (1) for ISO20. In the case of X-rays, owing to the *Q*-dependence of the atomic form factor, *f*(*Q*), the weighting factors cannot be expressed as constants. Therefore, in Eq. (8), the weighting factors are calculated using the atomic numbers,

8 $$\begin{aligned} S \left( Q \right) = & 0.509S_{{{\text{In}} - {\text{In}}}} \left( Q \right) + 0.334S_{{{\text{In}} - {\text{O}}}} \left( Q \right) + 0.075S_{{{\text{In}} - {\text{Si}}}} \left( Q \right) \\ & + 0.055S_{{{\text{O}} - {\text{O}}}} \left( Q \right) + 0.025S_{{{\text{Si}} - {\text{O}}}} \left( Q \right) + 0.003S_{{{\text{Si}} - {\text{Si}}}} \left( Q \right), \\ \end{aligned}$$

The weighting factors for the In–Si, Si–O, and Si–Si correlations were very small compared to those for the In–In or In–O correlations. Therefore, our data did not show clear Si–In, Si–O, or Si–Si peaks. We calculated the estimated density values of amorphous ISO2, 7, 11, and 20 samples from the slope of *G*(*r*) in the range of 0–1.30 Å (Table 1), and we utilized these values for the MD–RMC simulations^46^.

### Structural modeling

**Fig. 2:**
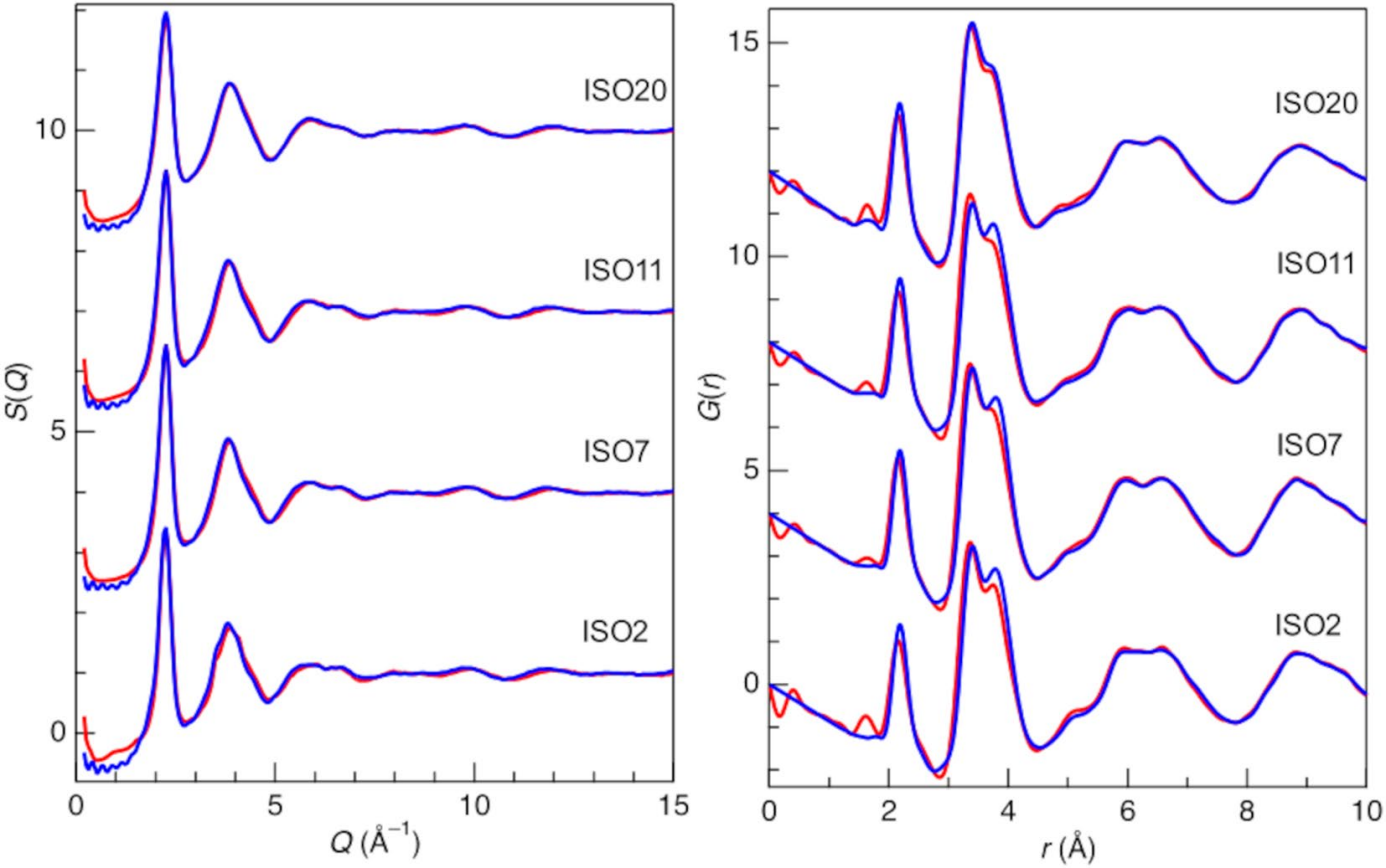
X-ray total structure factors, *S*(*Q*), and reduced pair distribution functions, *G*(*r*), of pristine ISO together with the results of CMD–RMC modeling. Red line, experimental data; blue line, CMD–RMC model.

In this study, we only developed structural models for pristine ISO2, 7, 11, and 20 because our objective was to elucidate the thermal stability of Si-doped amorphous ISO. The structural modeling of pristine ISO2, 7, 11, and 20 was conducted using CMD simulations (Fig. S2). While good agreement was found between the experimental and simulated *S*(*Q*) data, the simulation data of *G*(*r*) beyond 5.00 Å shifted to higher *r* compared to the experimental data. It is suggested that this discrepancy arose from the force field potentials, as the potential of Utsuno et al. was adjusted using a density value of 7.10 g/cm^3^ for amorphous In_2_O_3_, which is higher than the estimated density of our samples (approximately 6.00 g/cm^3^). Therefore, the CMD simulation was employed to make an initial configuration for the RMC refinement. Figure 2 shows the total structure factors, *S*(*Q*), and the reduced pair distribution functions, *G*(*r*), for the ISO2, 7, 11, and 20 samples. The agreement between the experimental and CMD–RMC data was significantly improved compared to the initial CMD model. Accordingly, we used the CMD–RMC model for further analysis.

All the *S*(*Q*) of ISO exhibited nearly identical peaks, notably two prominent peaks at approximately *Q* = 2.25 Å^−1^ and 3.85 Å^– 1^. A conventional oxide SiO_2_ glass showed two distinct peaks at approximately *Q*_1_ ~ 1.50 Å^– 1^ (first sharp diffraction peak) and *Q*_3_ ~ 5.20 Å^– 1^ in the *S*(*Q*), along with a significant oscillation at high *Q* attributed to the formation of SiO_4_ tetrahedra^47^. Although both ISO and SiO_2_ exhibited similar peaks, the first peak of ISO appeared sharper than that of SiO_2_. Intriguingly, the *S*(*Q*) of ISO resembles those of Zr_70_Cu_30_ metallic glass and liquid Hg^48^. One notable similarity is the high packing density, which indicates that ISO is more similar to Zr_70_Cu_30_ metallic glass and the liquid Hg structure than to SiO_2_ glass.

### Partial structural and short-range structural analysis of CMD–RMC models

**Fig. 3:**
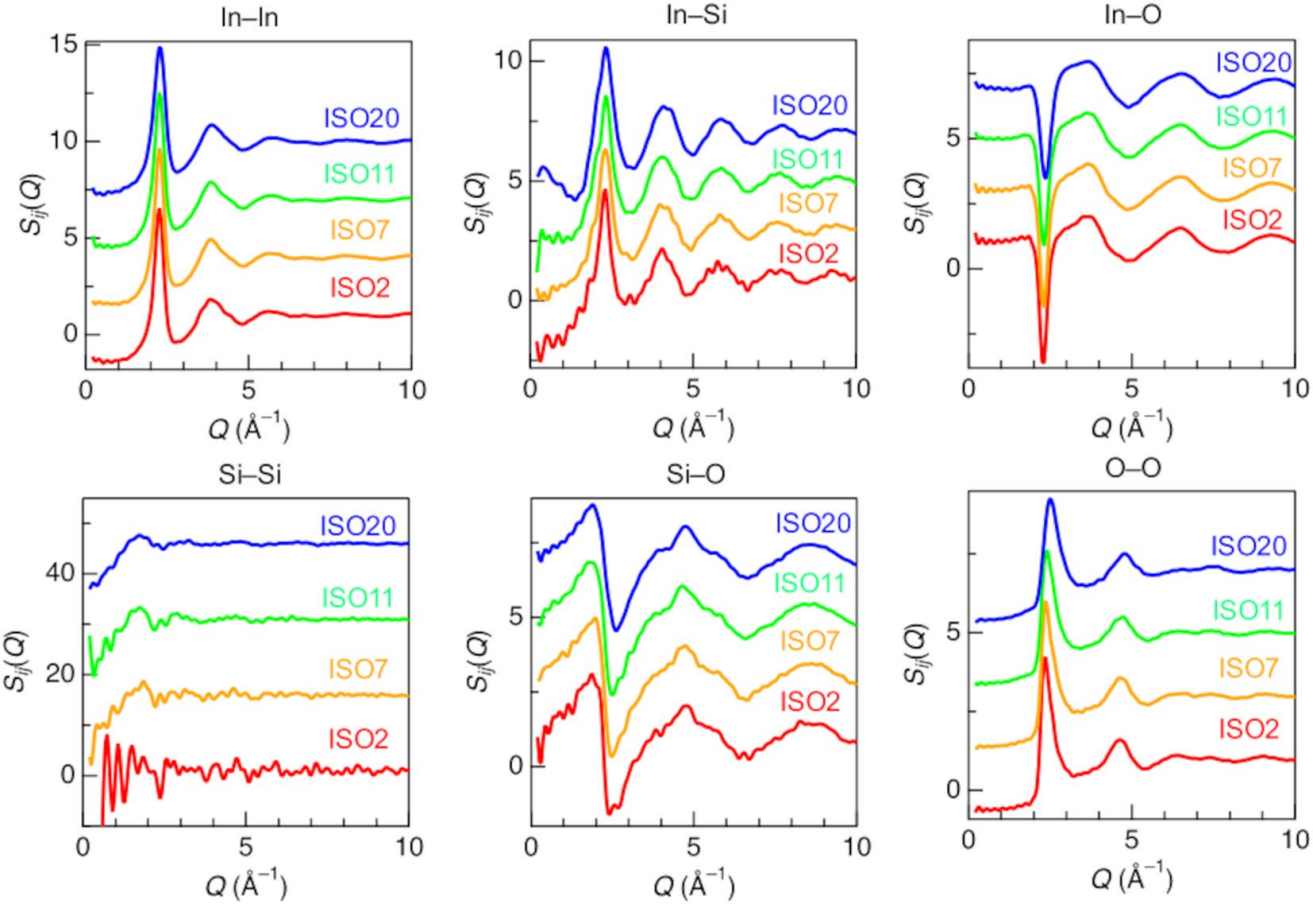
Partial structure factors, *S*_*ij*_(*Q*), of pristine ISO2, 7, 11, and 20. Red line, ISO2; orange line, ISO7; green line, ISO11; blue line, ISO20.

Figure 3 shows the partial structure factors, *S*_*ij*_(*Q*), of ISO2, 7, 11, and 20. Notably, the Si–Si correlation does not clearly exhibit peaks owing to the small fraction of Si atoms. The cation–oxygen *S*_*ij*_(*Q*) (*S*_In–O_(*Q*) and *S*_Si–O_(*Q*)) exhibits a negative peak at approximately *Q* ~ 2.30 Å, while the cation–cation *S*_*ij*_(*Q*) (*S*_In–In_(*Q*) and *S*_In–Si_(*Q*)) and *S*_O–O_(*Q*) exhibit a positive peak. The negative peaks in the cation–oxygen correlations indicate that the cation was located at the center of the oxygen polyhedron. In contrast, Yu reported^49^ a glass structure of BaTi_2_O_5_ showing positive cation–oxygen correlation peaks because the Ba atoms occupy off-center sites in the Ba–O polyhedra. In addition, the peak observed at approximately *Q* ~ 2.36 Å^– 1^ in the *S*_OO_(*Q*) shifts to a higher *Q* region with the addition of SiO_2_, which attribute to density changes.

**Fig. 4:**
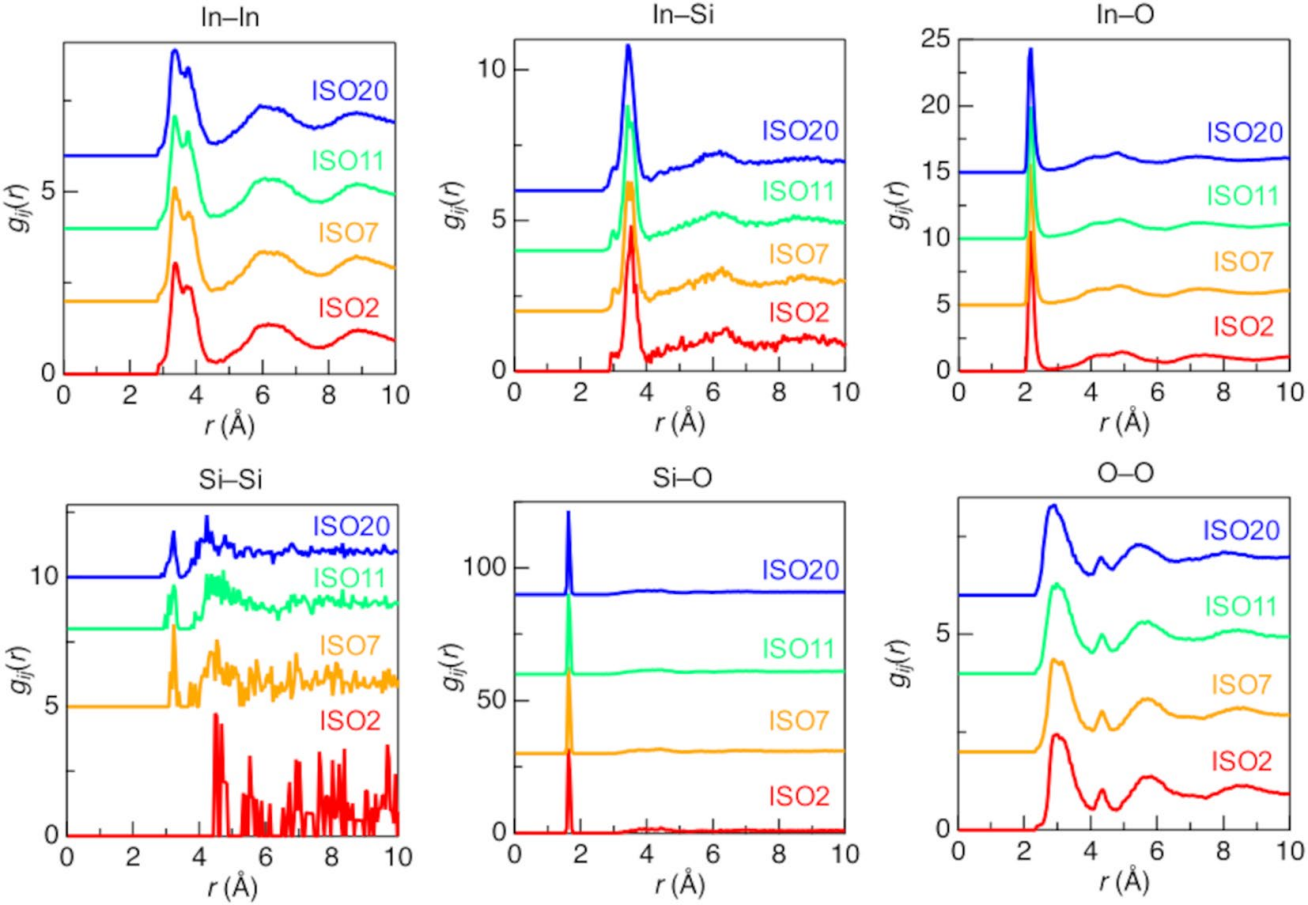
Partial pair distribution functions, *g*_*ij*_(*r*), of pristine ISO2, 7, 11, and 20. Red line, ISO2; orange line, ISO7; green line, ISO11; blue line, ISO20.

**Fig. 5:**
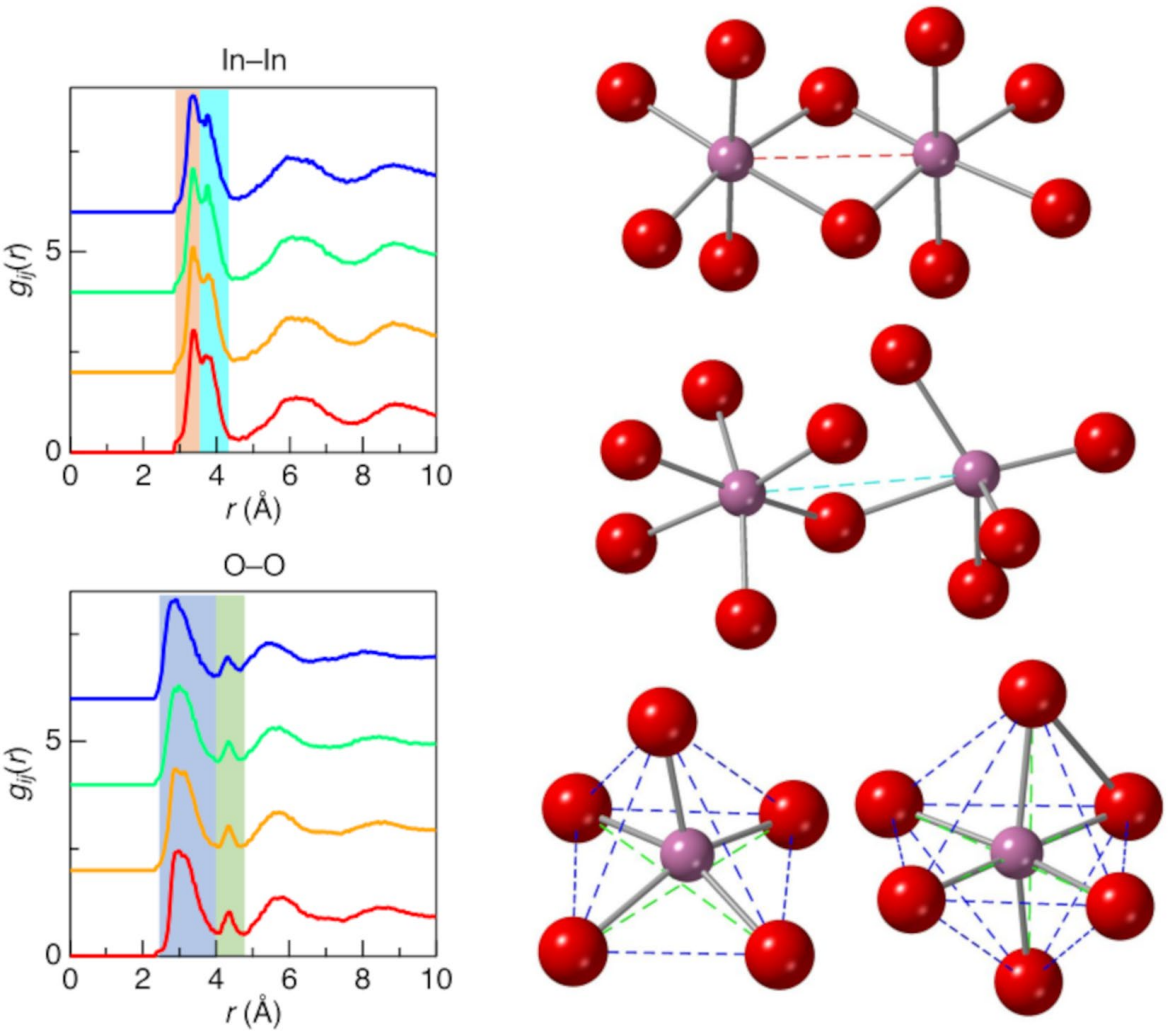
Comparison between the partial pair distribution function, *g*_*ij*_(*r*), of In–In and O–O correlation and illustration of InO_*x*_–InO_*x*_ polyhedral connectivity. The red region and dotted line indicates the edge-sharing In–In interatomic distance, the cyan region and dotted line indicates the corner-sharing In–In interatomic distance, the blue region and dotted line indicates the nearest neighbor O–O distance, and the green region and dotted line indicates the diagonal O–O distance.

Figure 4 shows the partial pair distribution functions, *g*_*ij*_(*r*), for ISO2, 7, 11, and 20 from the CMD–RMC modeling. No significant differences were observed with the addition of SiO_2_. This result is crucial for understanding the thermal stability of ISO because it suggests that Si doping does not have a significant effect on structural changes. In other words, the structural changes induced by Si doping are not the origin of the ISO thermal stability. The features observed for *g*_In–In_(*r*) and *g*_O–O_(*r*) are particularly interesting. The *g*_In–In_(*r*) from 2.50 to 4.00 Å shows two peaks, while *g*_O–O_(*r*) exhibits tiny peaks at approximately 4.20 Å. Figure 5 illustrates the polyhedral connectivity of InO_*x*_–InO_*x*_ with corner and edge sharing. The *g*_In–In_(*r*) at approximately 3.20 Å and 3.90 Å indicate corner and edge-sharing of InO_*x*_–InO_*x*_. In addition, the small peaks of *g*_O–O_(*r*) indicate an oxygen–oxygen correlation on the diagonal of the oxygen polyhedron.

### Topological analysis

**Fig. 6:**
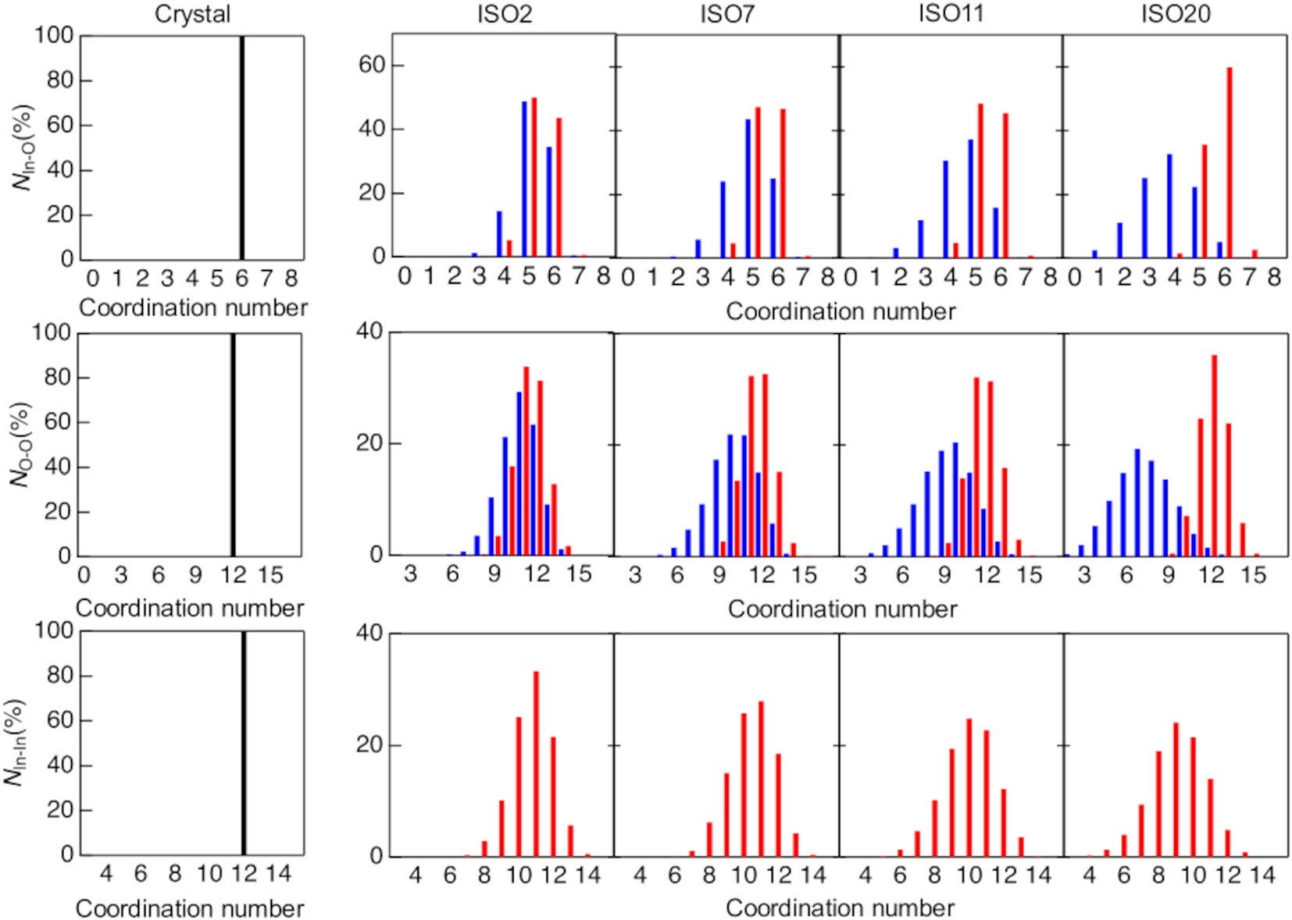
Coordination number distributions of crystalline In_2_O_3_ and pristine ISO2, 7, 11, and 20. Black bar, crystalline; red line, ISO with SiO_4_ tetrahedra; blue line, ISO without SiO_4_ tetrahedra.

**Fig. 7:**
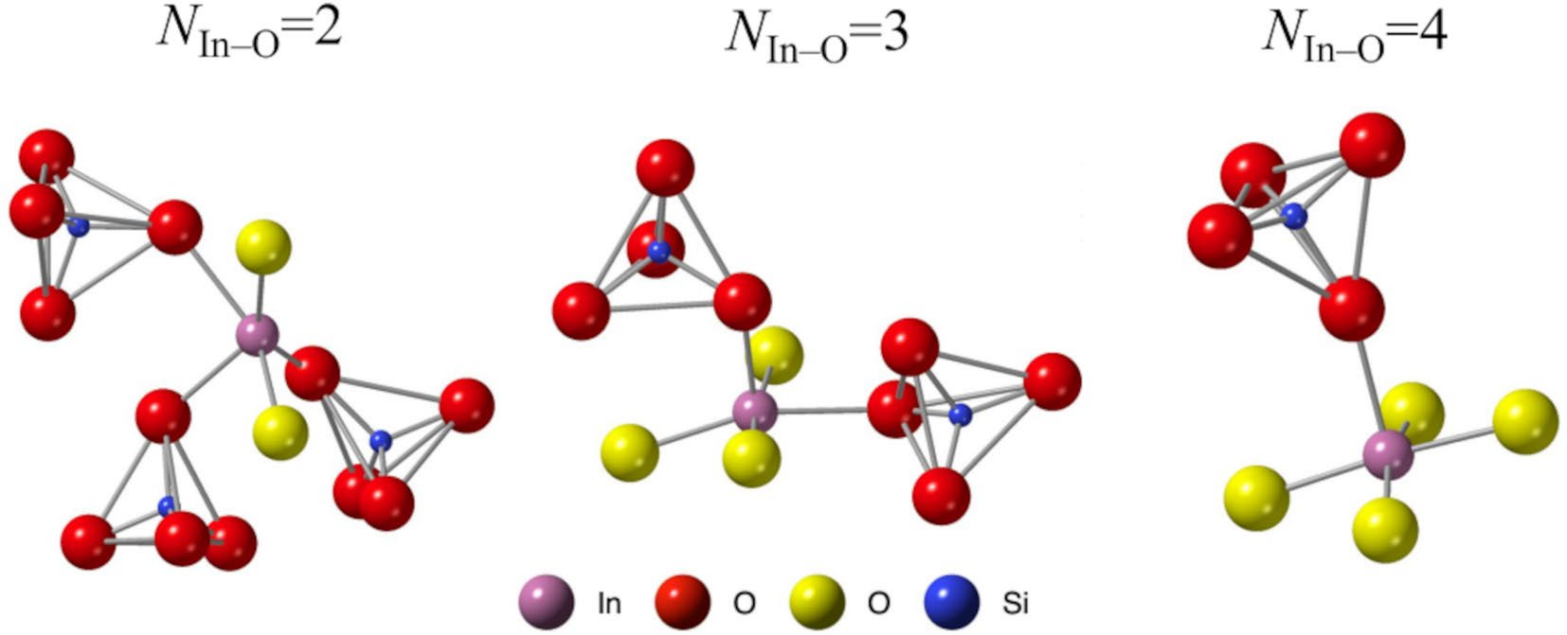
Typical polyhedral arrangements of a InO_5_ polyhedron and SiO_4_ tetrahedra.

Figure 6 shows the In–In, In–O, and O–O coordination number distributions, and Table 3 presents the average coordination numbers for the ISO. Notably, the In–O and O–O data include two types of analyses, one of which excludes SiO_4_ tetrahedra from the structural models (denoted by blue bars), as they are not relevant to the crystallization of ISO. Figure 7 shows the InO_5_ polyhedron and SiO_4_ tetrahedra. We counted only the bridging oxygens of –In–O–In–, excluding the oxygen atoms of the SiO_4_ tetrahedra. The coordination number of the In–O-containing SiO_4_ tetrahedra approaches that of the crystal structure (*N*_In–O_ = 6) with increasing Si content. This implies that the addition of SiO_2_ facilitates crystallization but hinders the thermal stability of ISO. In contrast, in the *N*_In–O_ without SiO_4_ tetrahedra, the addition of Si led to broader peaks and decreased coordination numbers. This phenomenon is similar to that observed in amorphous materials^23,26^.

**Fig. 8:**
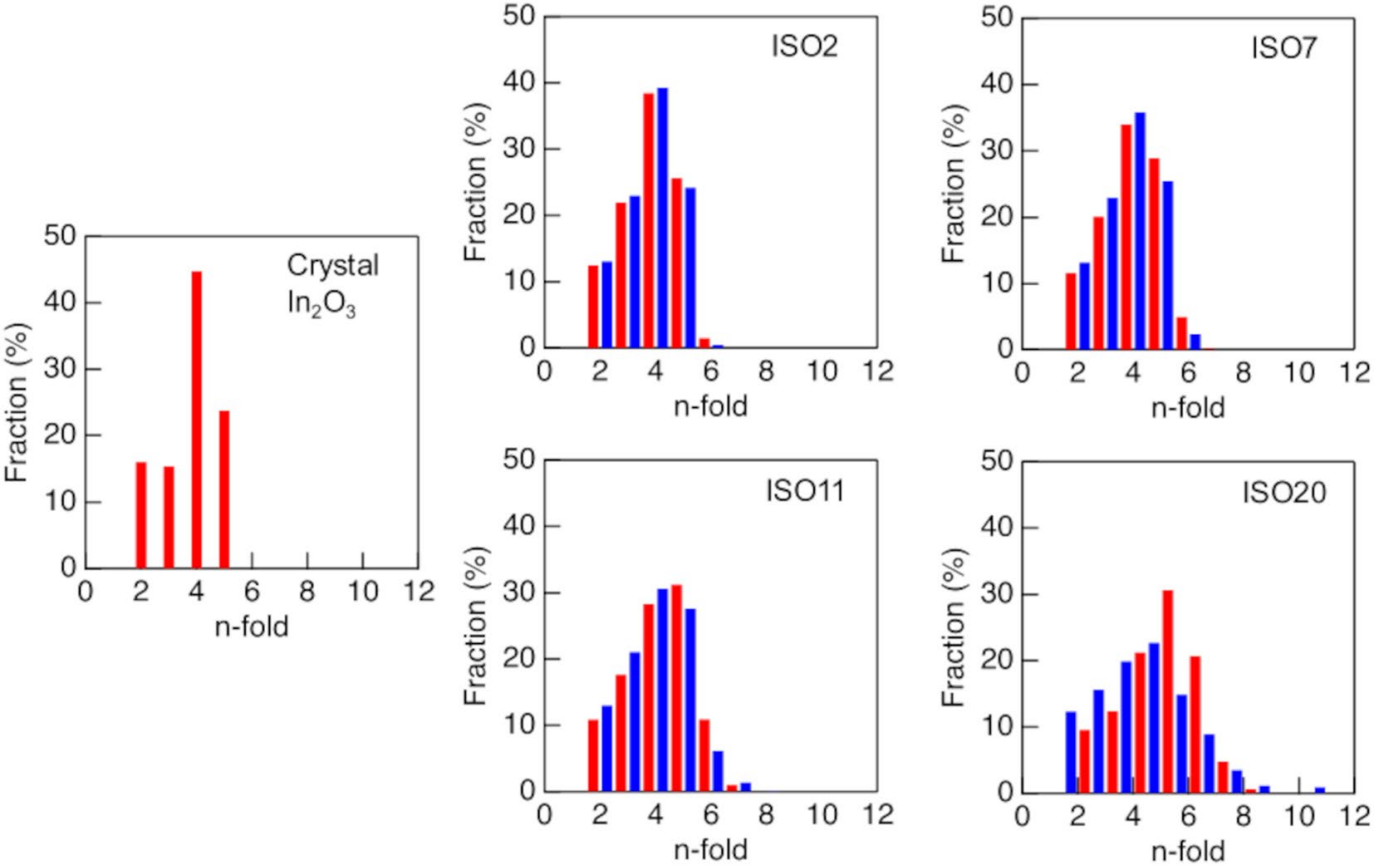
Kings ring size distribution for crystalline In_2_O_3_ and pristine ISO2, 7, 11, and 20. Red line, crystalline In_2_O_3_ and ISO with SiO_4_ tetrahedra; blue line, ISO without SiO_4_ tetrahedra.

Figure 8 shows the ring size distribution of crystalline In_2_O_3_ and ISO2, 7, 11, and 20. Four-fold rings were predominant in crystalline In_2_O_3_. Cooper reported that SiO_2_ glass exhibits a wide distribution of rings, including large-fold rings^50^. Therefore, the dominance of four-fold rings in crystalline In_2_O_3_ indicates that this material is densely packed. All ring size distributions, except that for ISO20, show broad distributions owing to Si doping, but no significant differences between those with and without SiO_4_ tetrahedra were observed. ISO20, with and without SiO_4_, shows small differences, but both sets of data exhibit broad peaks, and the number of large rings increases. These results indicate that the thermal stability of ISO cannot be determined from structural information related to the intermediate- and long-range orders.

Table 4 summarizes the connectivity between InO_*x*_–SiO_4_ and InO_*x*_–InO_*x*_. All ISOs were within the corner-sharing motif for InO_*x*_–SiO_4_, although small fractions of edge-sharing polyhedra were observed. SiO_4_ tetrahedra are well-known 100% corner-sharing in SiO_2_ glass, which is a typical glass-forming oxide, and SiO_4_ of ISO exhibits similar behavior. The InO_*x*_–InO_*x*_ connectivities with SiO_4_ in ISO show a corner-sharing motif, but the fraction of edge-sharing polyhedra increases. In particular, InO_*x*_–InO_*x*_ for *c*-In_2_O_3_ exhibited 50% edge sharing. Koyama et al.^21^ reported that Er_2_O_3_ liquid, which is classified as a non-glass-forming oxide, exhibits a large fraction of edge-sharing ErO_*n*_ polyhedra. Based on this behavior, we conclude that the fraction of edge-sharing InO_*x*_–InO_*x*_ is the most crucial factor in understanding the crystallization of ISO. The fraction of edge-sharing InO_*x*_–InO_*x*_ with SiO_4_ shows almost the same value, suggesting that changing the number of Si atoms has no effect on the thermal stability of ISO. To reconsider this contradiction, we compared the fractions of edge-sharing InO_*x*_–InO_*x*_ with and without SiO_4_. The fraction of edge-sharing InO_*x*_–InO_*x*_ without SiO_4_ decreased with Si doping. The dissociation energy of the Si–O bond was 799 kJ/mol, whereas that of the In–O bond was 346 kJ/mol^51^, indicating that the Si–O bond had stronger connectivity than the In–O bond. This suggests that the SiO_4_ tetrahedra of ISO are not broken down by the In–O bonds. Based on these results, we interpret the thermal stability of ISO to be derived from the strong covalent bonds of SiO_4_ tetrahedra maintaining their configuration, and the SiO_4_ tetrahedra prevent the formation of edge-sharing InO_*x*_–InO_*x*_ during annealing treatment.

**Table 3:** Average coordination numbers of crystalline In_2_O_3_ and pristine ISO2, 7, 11, and 20 with and without SiO_4_ tetrahedra.

	Crystal	ISO2	ISO7	ISO11	ISO20
With SiO_4_					
In–O	6.00	5.39	5.44	5.42	5.64
O–O	12.00	11.39	11.52	11.54	11.97
In–In	12.00	10.79	10.48	9.98	9.11
Si–O	–	4.00	4.00	4.00	4.00
Without SiO_4_					
In–O	6.00	5.19	4.89	4.51	3.77
O–O	12.00	10.89	10.12	9.28	7.36
In–In	12.00	10.79	10.48	9.98	9.13

**Table 4:** Polyhedral connectivity in crystalline In_2_O_3_ and pristine ISO2, 7, 11, and 20 with and without SiO_4_ tetrahedra.

	Corner	Edge	Face
InO_x_–SiO_4_ with SiO_4_			
ISO2	94.4	5.6	0.0
ISO7	95.3	4.7	0.0
ISO11	95.7	4.3	0.0
ISO20	94.6	5.4	0.0
InO_*x*_–InO_*x*_ with SiO_4_			
Crystal	50.0	50.0	0
ISO2	69.0	30.0	1.1
ISO7	68.6	30.3	1.1
ISO11	69.1	29.6	1.3
ISO20	65.3	32.8	1.9
InO_*x*_–InO_*x*_ without SiO_4_			
ISO2	70.1	28.9	1.0
ISO7	71.7	27.5	0.8
ISO11	73.9	25.2	0.8
ISO20	76.4	22.9	0.7

## Conclusions

In this article, we discuss the relationship between the atomic structure and thermal stability of Si-doped amorphous In_2_O_3_. X-ray *S*(*Q*) and *G*(*r*) clarify the atomic distances, revealing that pristine samples of ISO2, 7, 11, and 20 exhibit an amorphous structure, whereas ISO, except ISO20, crystalizes after annealing at 600 °C. Moreover, the atomic correlation peaks of the Si-doped sample did not significantly change in real space. Combining CMD and RMC in a simulation allows us to perform a more precise structural modeling of the ISO compared to the CMD simulation alone. The *S*(*Q*) of the ISO samples is more similar to those of Zr_70_Cu_30_ metallic glass and Hg liquid than that of SiO_2_ glass because of the densely packed structure in ISO. The In–O coordination number distribution broadened in the atomic configuration of the ISO without SiO_4_ tetrahedra with increasing Si content. Analysis of the polyhedral connectivity revealed that the majority of the connectivity of the SiO_4_ tetrahedra is corner-sharing with the InO_*x*_ polyhedra. In addition, the edge-sharing of InO_*x*_–InO_*x*_ without SiO_4_ tetrahedra decreases with increasing Si doping. We conclude that the maintenance of the amorphous structure of Si-doped ISO is attributed to the tolerance of SiO_4_ tetrahedra formed by covalent bonds, preventing the formation of edge-sharing InO_*x*_–InO_*x*_ during heating treatment.

In this study, the structure of amorphous In_2_O_3_ was constructed using CMD simulations and then refined via RMC simulation to yield a model consistent with the experimental data. Recently, advances in high-precision methods of constructing potentials, including machine learning force fields (ML-FFs),^52^ which enable the application of consistent potentials across different compositions, have been reported, and these methods may be effective in physically reasonable structural analyses. However, constructing the potential was beyond the scope of this study, and thus, RMC was adopted, focusing on practicality and consistency with the experimental results. In the future, improvements in CMD potentials and the introduction of ML-FFs should enable analyses using potentials that are consistent across all compositions.

These findings contribute to a better understanding of the role of Si doping in controlling the structural changes and properties of amorphous oxides. We believe that our findings will be crucial for various applications in optoelectronics and solid-state devices.

## Supplementary Information

Below is the link to the electronic supplementary material.

Supplementary Material 1

## Data Availability

Data is provided within the manuscript or supplementary information files. The datasets used and/or analyzed during the current study available from the corresponding author on reasonable request.
