# Supplementary material for: Structural analysis of Si-doped amorphous In2O3 based on quantum beam measurements and computer simulations
Source: Sci Rep. 2025 Oct 21;15:36662. doi: 10.1038/s41598-025-20384-0 (PMC12540856; doi:10.1038/s41598-025-20384-0)
Supplement: Supplementary file 1 — Supplementary Material 1 [file 41598_2025_20384_MOESM1_ESM.docx]

**Structural Analysis of Si-Doped Amorphous In_2_O_3_ Based on Quantum Beam Measurements and Computer Simulations**

by Yuta Shuseki et al.

|  | Density (g/cm^3^) |
| --- | --- |
| Experiment | 2.20 |
| From *G*(*r*) | 2.27 |

Fig. S1 Elucidation of the error in estimated density using SiO_2_ glass.

**
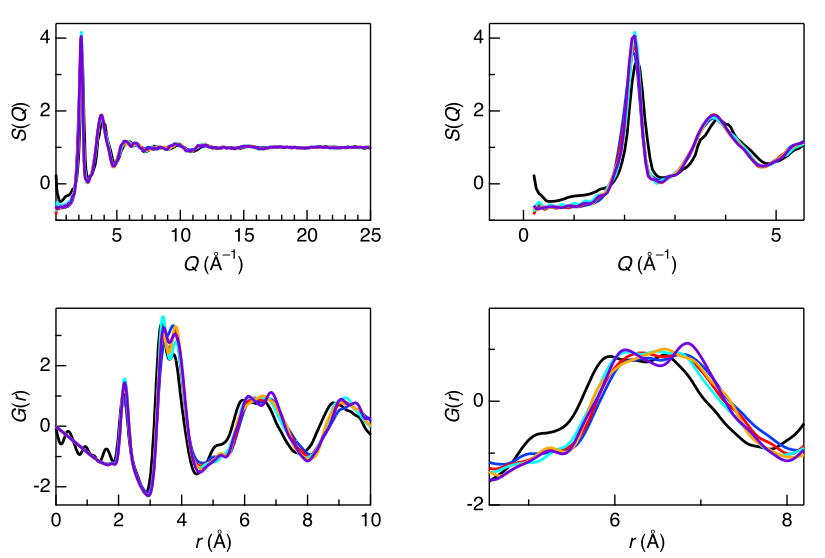
**

Fig. S2 X-ray total structure factors, *S*(*Q*), of pristine ISO together with the result of CMD modeling. Black line, experimental data (In_2_O_3_);

Red line, Utsuno *et al.*’s potential^37)^ (condition; ensemble: NVT, melt: 10 ps at 10000 K, quench rate: 970 K/ps from 10000K to 300K, equilibrium: 10 ps at 300 K, total atom number: 10000).

Green line, Utsuno’s potential^37)^ (condition; ensemble: NVT, melt: 1000 ps at 10000 K, quench rate: 9.7 K/ps from 10000K to 300K, equilibrium: 1000 ps at 300 K, total atom number: 5000).

Blue line, Utsuno’s potential^37)^ (condition; ensemble: NVT, melt: 10000 ps at 10000 K, quench rate: 0.97 K/ps from 10000K to 300K, equilibrium: 10000 ps at 300 K, total atom number: 5000).

Orange line, Ide’s potential^39)^ (condition; ensemble: NVT, melt: 1000 ps at 10000 K, quench rate: 5K/ps from 10000K to 300K, equilibrium: 1000 ps at 300 K, total atom number: 10000).

Cyan line, Ide’s potential^39)^ (condition; ensemble: NVT, melt: 10000 ps at 10000 K, quench rate: 0.97K/ps from 10000K to 300K, equilibrium: 10000 ps at 300 K, total atom number: 10000).

Fig. S3 X-ray total structure factors, *S*(*Q*), and reduced pair distribution functions, *G*(*r*), of ISO2, 7, 11, and 20. Blue line, MD–RMC simulation models; red line, MD simulation models.

Table S1 Average coordination numbers of ISO0, 2, 7, 11, and 20 obtained from MD and MD–RMC simulations.

| MD | In–In | In–O | O–O |
| --- | --- | --- | --- |
| ISO0 | 11.56 | 5.46 | 11.56 |
| ISO2 | 11.2 | 5.44 | 11.45 |
| ISO7 | 10.78 | 5.49 | 11.69 |
| ISO11 | 10.25 | 5.47 | 11.61 |
| ISO20 | 9.33 | 5.68 | 12.06 |
|  |  |  |  |
| MD–RMC | In–In | In–O | O–O |
| ISO0 | - | - | - |
| ISO2 | 10.79 | 5.39 | 11.39 |
| ISO7 | 10.48 | 5.44 | 11.52 |
| ISO11 | 9.98 | 5.42 | 11.54 |
| ISO20 | 9.11 | 5.64 | 11.97 |

Fig. S4 Reduced pair distribution functions, *G*(*r*), of crystalline In_2_O_3_ calculated using PDFgui. Red line, annealed In_2_O_3_ (ISO0); bule line, cubic In_2_O_3_; green line, trigonal In_2_O_3_; light bule, orthorhombic In_2_O_3_.
